# SIRT3 is attenuated in systemic sclerosis skin and lungs, and its pharmacologic activation mitigates organ fibrosis

**DOI:** 10.18632/oncotarget.12504

**Published:** 2016-10-06

**Authors:** Kaname Akamata, Jun Wei, Mitra Bhattacharyya, Paul Cheresh, Michael Y. Bonner, Jack L. Arbiser, Kirtee Raparia, Mahesh P. Gupta, David W. Kamp, John Varga

**Affiliations:** ^1^ Division of Rheumatology, Feinberg School of Medicine, Northwestern University, Chicago, IL, USA; ^2^ Division of Pulmonary & Critical Care Medicine, Feinberg School of Medicine, Northwestern University, Chicago, IL, USA; ^3^ Department of Dermatology, Emory University School of Medicine, Atlanta, GA, USA; ^4^ Atlanta Veterans Administration Medical Center and Winship Cancer, Atlanta, GA, USA; ^5^ Department of Pathology, Northwestern University, Chicago, IL, USA; ^6^ Department of Surgery, University of Chicago, Chicago, IL, USA; ^7^ Jesse Brown VA Medical Center, Chicago, IL, USA

**Keywords:** fibrosis, SIRT3, TGF-β, myofibroblast, ROS, Pathology Section

## Abstract

Constitutive fibroblast activation is responsible for organ fibrosis in fibrotic disorders including systemic sclerosis (SSc), but the underlying mechanisms are not fully understood, and effective therapies are lacking. We investigated the expression of the mitochondrial deacetylase sirtuin 3 (SIRT3) and its modulation by hexafluoro, a novel fluorinated synthetic honokiol analogue, in the context of fibrosis. We find that augmenting cellular SIRT3 by forced expression in normal lung and skin fibroblasts, or by hexafluoro treatment, blocked intracellular TGF-ß signaling and fibrotic responses, and mitigated the activated phenotype of SSc fibroblasts. Moreover, hexafluoro attenuated mitochondrial and cytosolic reactive oxygen species (ROS) accumulation in TGF-β-treated fibroblasts. Remarkably, we found that the expression of SIRT3 was significantly reduced in SSc skin biopsies and explanted fibroblasts, and was suppressed by TGF-β treatment in normal fibroblasts. Moreover, tissue levels of acetylated MnSOD, a sensitive marker of reduced SIRT3 activity, were dramatically enhanced in lesional skin and lung biopsies from SSc patients. Mice treated with hexafluoro showed substantial attenuation of bleomycin-induced fibrosis in the lung and skin. Our findings reveal a cell-autonomous function for SIRT3 in modulating fibrotic responses, and demonstrate the ability of a novel pharmacological SIRT3 agonist to attenuate fibrosis *in vitro* and *in vivo*. In light of the impaired expression and activity of SIRT3 associated with organ fibrosis in SSc, pharmacological approaches for augmenting SIRT3 might have therapeutic potential.

## INTRODUCTION

Fibrosis, defined by the excessive accumulation of the extracellular matrix (ECM) in inflamed tissue, leads to permanent scarring and organ malfunction [1]. Systemic sclerosis (SSc) is characterized by widespread fibrosis, accompanied by autoimmunity and vasculopathy [2]. Greater than 50% of patients with SSc die, or develop organ failure, within ten years, with mortality due in large part to fibrosis in the lungs [3]. Fibrosis is caused by activation of myofibroblasts triggered and then perpetuated by transforming growth factor-β (TGF-β) and related growth factors, cytokines, peptides, hypoxia and stiffening of the matrix [4]. Transforming growth factor-β also stimulates production of reactive oxygen species (ROS) from mitochondrial and cytosolic enzymes, which leads to cellular oxidative stress and directly contributes to myofibroblast differentiation and fibrosis progression [5, 6].

Sirtuins (SIRTs), the mammalian orthologs of yeast silent mating-type information regulator 2 (Sir2), are NAD-dependent class III deacetylases with pleiotropic effects on metabolism, cell survival, cancer, processes of aging, and calorie restriction-mediated longevity in organisms ranging from yeast to human [7–9]. The most extensively studied member of the family, SIRT1, is principally nuclear, while SIRT3 is located withinmitochondriaand implicated in mitochondrial homeostasis [10, 11]. Fibroblasts lacking SIRT3 exhibit aberrant glucose metabolism and generate increased levels of ROS when exposed to oxidative stress, genotoxic stress, and ionizing radiation [12]. Using genome-wide transcriptome profiling, we previously showed that treatment of explanted healthy and SSc fibroblasts with TGF-ß resulted in down-regulation of SIRT3 expression [13]. Of interest, a recent study showed that pharmacologic activation or forced expression of SIRT3 attenuated alpha-smooth muscle actin (α-SMA) and collagen gene expressions in a mouse model of cardiac hypertrophy [14].

Mitochondria are a significant source of ROS such as the superoxide radical (O_2_^−^) that can react with DNA and other biomolecules to induce mutagenesis, senescence, and ultimately cell death [15]. The primary mitochondrial mechanism for maintaining physiological O_2_^−^ involves manganese superoxide dismutase (MnSOD) [16]. Acetylation of mitochondrial MnSOD catalyzed by SIRT3 plays a pivotal role in modulating its enzymatic activity, and down-regulation of SIRT3 is associated with enhanced levels of acetylated MnSOD in tissues [17–19].

Honokiol (HNK) [2-(4-hydroxy-3-prop-2-enyl-phenyl)-4-prop-2-enyl-phenol], a small molecular weight biphenolic compound derived from the magnolia tree bark, has anti-thrombotic, anti-inflammatory, anti-tumor, cytoprotective and anti-oxidative activities, suggesting a potential therapeutic utility in a variety of chronic conditions [20–23]. A recent study showed that HNK induced SIRT3 in cardiomyocytes *in vitro* and *in vivo* [14]. In an effort to generate more lipophilic HNK derivatives with enhanced drug-like properties, hexafluoro (bis-trifluoromethyl-bis-(4-hydroxy-3-allylphenyl) methane) was synthesized and shown to have activity against vemurafenib-resistant melanoma cells in vivo [24].

Little is currently known regarding the expression and function of SIRT3 in fibroblasts, or their potential role in fibrosis in SSc. Moreover, the modulation of SIRT3 by HNK derivatives in this context is not understood. Our present results show impaired expression and activity of SIRT3 in skin and lung biopsies from patients with SSc. In normal fibroblasts, TGF-ß treatment caused down-regulation of cellular SIRT3, while hexafluoro rescued SIRT3 expression in these cells. Moreover, hexafluoro abrogated TGF-β-induced stimulation of fibrotic gene expression, myofibroblast differentiation, and cellular ROS production in normal fibroblasts. *In vivo*, hexafluoro mitigated bleomycin-induced lung and skin fibrosis in the mouse. These results implicate aberrant SIRT3 function in the pathogenesis of SSc, and suggest that pharmacological enhancement of SIRT3 may represent a promising novel therapeutic approach.

## RESULTS

### SIRT3 negatively regulates fibrotic responses

**Figure 1:**
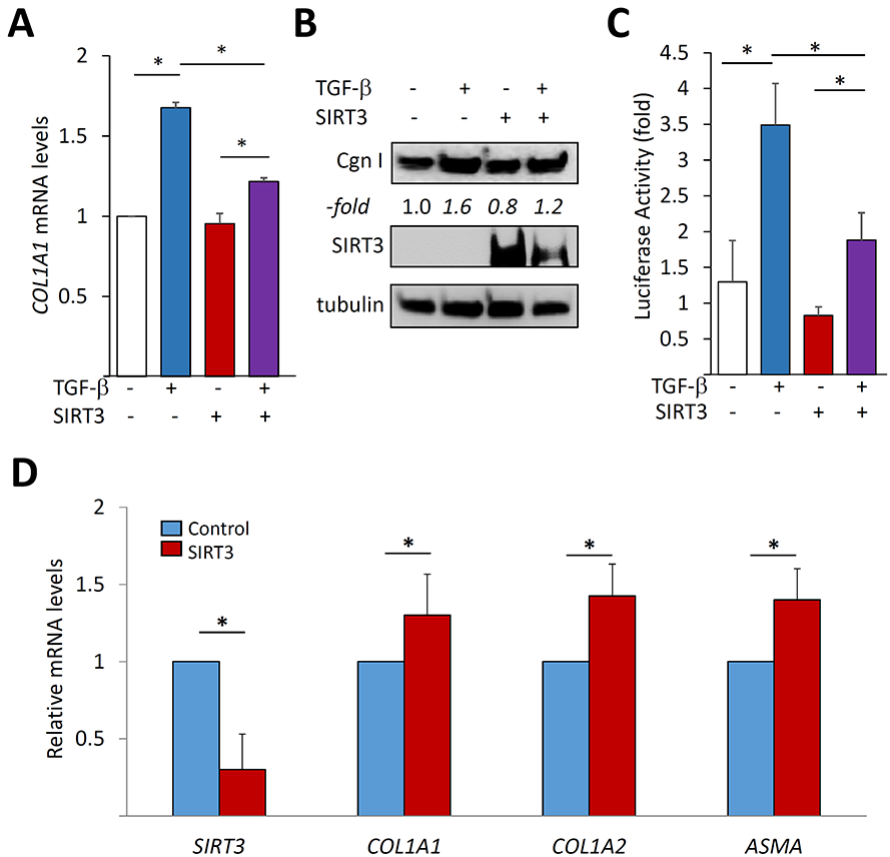
SIRT3 negatively regulates fibrotic responses **A.**-**C.** Normal adult lung fibroblasts transfected with SIRT3 or empty vector were incubated with TGF-β2 (10 ng/ml) for 24 h. A. Results of real-time qPCR normalized with *GAPDH* are means ± SD of triplicate determinations from an experiment representative of three. *p < 0.05. **B.** Whole cell lysates were examined by Western analysis. Representative blots from an experiment representative of three; -fold change in band intensities normalized with tubulin shown below. Cgn I, Type I collagen. **C.** Fibroblasts were co-transfected [SBE]_4_-luc along with SIRT3 or empty vector. Whole cell lysates were analyzed for their luciferase activities. Results are the means ± SD of triplicate experiments. *p < 0.05. **D.** Fibroblasts were transiently transfected with *SIRT3* siRNA or scrambled (control) siRNA and incubated for 48 h. Results of qRT-PCR normalized with *GAPDH* are means ± SD of triplicate determinations from an experiment representative of three. *p < 0.05.

Excessive mitochondrial ROS production is implicated in the pathogenesis of fibrosis in multiple organs [25, 26]. As a mitochondrial deacetylase, SIRT3 has a vital function in maintaining mitochondrial integrity and modulating oxidative stress [27]. To examine the potential role of SIRT3 in controlling fibrotic responses, we used gain-of-function and loss-of-function experiments. Ectopic expression of SIRT3 in normal lung fibroblasts was sufficient to suppress TGF-β-induced stimulation of collagen synthesis (Figure 1A and 1B). It was of interest to delineate the intracellular pathways targeted by SIRT3 in these cells, focusing on canonical Smad signaling, the principal mechanism for fibrotic signaling [4]. Transient transfection assays demonstrated that TGF-β stimulation of a Smad-responsive [SBE]_4_-luc reporter in fibroblasts was significantly reduced by SIRT3 overexpression (Figure 1C). Conversely, even partial down-regulation of cellular SIRT3 by RNAi in normal lung fibroblasts resulted in spontaneous elevation of multiple fibrotic genes (Figure 1D). Comparable results were seen using skin fibroblasts from healthy adult donors (Suppl. Fig 1). Collectively, these results indicate that in fibroblasts SIRT3 serves as a cell-autonomous negative regulator to constrain fibrotic gene expression.

### Hexafluoro enhances SIRT3 expression

**Figure 2:**
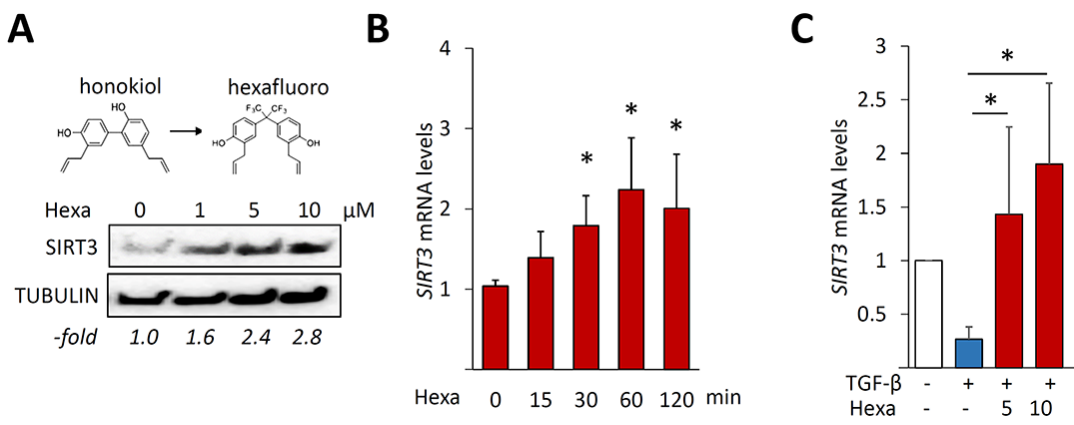
Hexafluoro stimulates SIRT3 expression Lung fibroblasts were incubated with hexafluoro (10 μM or indicated concentrations) for 24h or indicated periods, in the absence or presence of TGF-β2 (10 ng/ml and indicated times). **A.** Whole cell lysates were examined by Western analysis. Representative blots. – fold change in band intensities normalized with tubulin shown below. **B.**, **C.** Real-time qPCR results normalized with *GAPDH* are means ±SD of triplicate determinations from an experiment representative of three. *, *p* < 0.05.

In light of SIRT3's anti-fibrotic effects, we speculated that it may have a physiologic regulatory role in maintaining fibroblast homeostasis, and that failure of this regulatory function could contribute to pathologic fibrosis. To begin to explore this concept, we used a novel pharmacological analogue of honokiol, a naturally-occurring biphenolic compound derived from the magnolia tree bark that was recently shown to be a SIRT3 activator [24]. The novel honokiol derivative hexafluoro (Figure 2A, upper panel) was synthesized by adding bis-trifluoro methyl radicals to honokiol [24] with better lipophilic characteristics. Incubation of confluent fibroblasts with hexafluoro resulted in time- and dose-dependent augmentation of SIRT3, while no increase in SIRT1 was seen (Figure 2B and data not shown). Trypan blue dye exclusion and LDH assays showed that cell viability was unaffected by hexafluor in concentrations up to 10 μM of hexafluoro (data are not shown). Treatment of human lung fibroblasts with TGF-β caused a significant decrease in SIRT3 mRNA and protein levels (Figure 2C). Skin fibroblasts showed a comparable down-regulation of SIRT3 (Suppl. Figs. 2A and B, and data not shown). Remarkably, pre-incubation of the cultures with hexafluoro for 30 min rescued *SIRT3* mRNA expression even in the presence of TGF-β (Figure 2C).

### Hexafluoro blocks TGF-β-induced fibrotic responses

**Figure 3:**
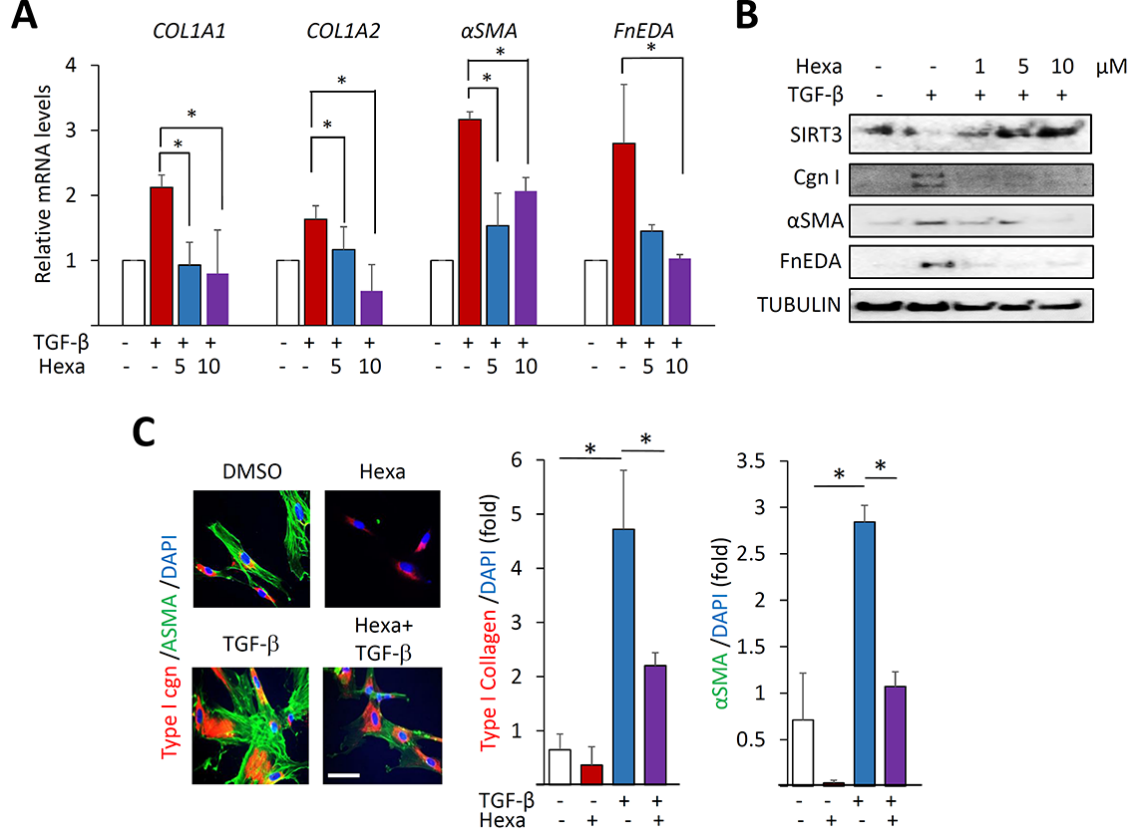
Hexafluoro attenuates TGF-β-induced fibrotic gene expression Lung fibroblasts were preincubated with hexafluoro (10 μM) for 30 min, followed by TGF-β2 (10 ng/ml) for 24 h. **A.** Relative mRNA levels were examined by real-time qPCR. Results normalized with *GAPDH* are means ±SD of triplicate determinations from a representative experiment of three. *, *p* < 0.05. **B.** Whole cell lysates were examined by Western analysis. Representative images. Cgn I, Type I collagen. **C.** Left, Confocal immunofluorescence using antibodies to αSMA (green) and Type I collagen (red). Bar = 10μm. Right, Quantification of immunofluorescence intensity. Results are the means ±SD of triplicate experiments.

**Figure 4:**
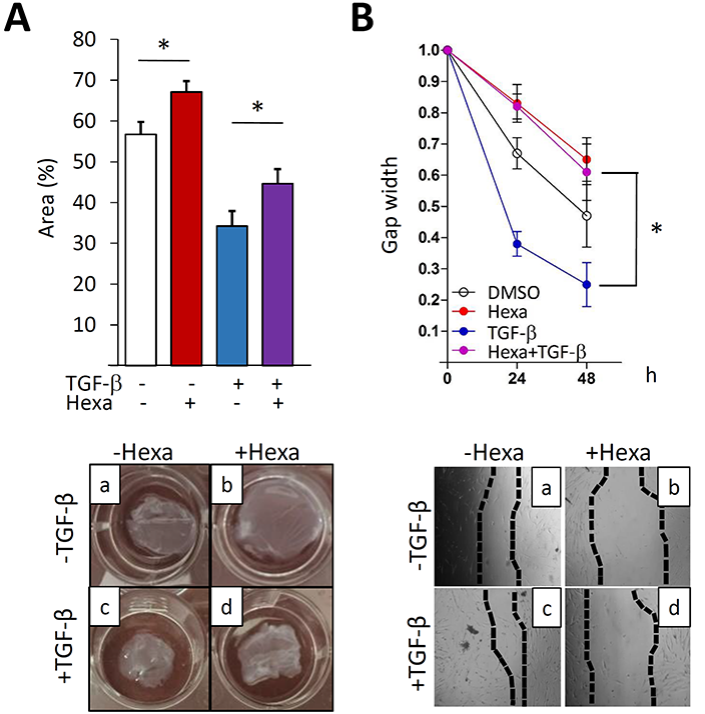
Hexafluoro attenuates TGF-β-induced cell contraction and migration Lung fibroblasts were pretreated with 10 μM hexafluoro for 30 min, followed by TGF-β2 (10 ng/ml) for further 72 (A) and 48 h (B). **A.** Gel contraction assays (*n* = 3). Representative images. Results are means ±SD of triplicate experiments. *p < 0.05. **B.** Wound healing assays (*n* = 3). Representative images. Results are means ±SD of triplicate experiments. *p < 0.05.

In order to further explore the modulation of fibroblast responses, confluent lung fibroblasts were pre-incubated with hexafluoro (10 μM) for 30 min prior to TGF-β (10 ng/ml), and harvested following a further 24 h incubation. Treatment with TGF-β induced a significant increase in the expression of fibrotic gene such as COL1A1, COL1A2, αSMA and fibronectin EDA (FN^EDA^), and in myofibroblast differentiation, as expected, while hexafluoro pretreatment dose-dependently suppressed these fibrotic responses accompanied by increase in levels of SIRT3 (Figure 3A-3C). Significantly, hexafluoro exerted comparable anti-fibrotic effects in skin fibroblasts (Suppl. Figs 2C, D). Moreover, abrogation of TGF-β–induced fibrotic responses by hexafluoro was seen even when hexafluoro added after TGF-β (Suppl. Fig 2E). To investigate the effects of hexafluoro on tissue remodeling, *in vitro* wound healing and collagen lattice gel contraction assays were performed [28]. Incubation with hexafluoro significantly attenuated TGF-β-induced stimulation of both fibroblast contractility (Figure 4A) and migration (Figure 4B). Collectively, these results indicate that hexafluoro potently inhibits the induction of fibrotic gene expression, myofibroblast differentiation, contraction and migration in normal fibroblasts.

### Hexafluoro mitigates ROS enhanced production and changes in mitochondrial membrane potential

**Figure 5:**
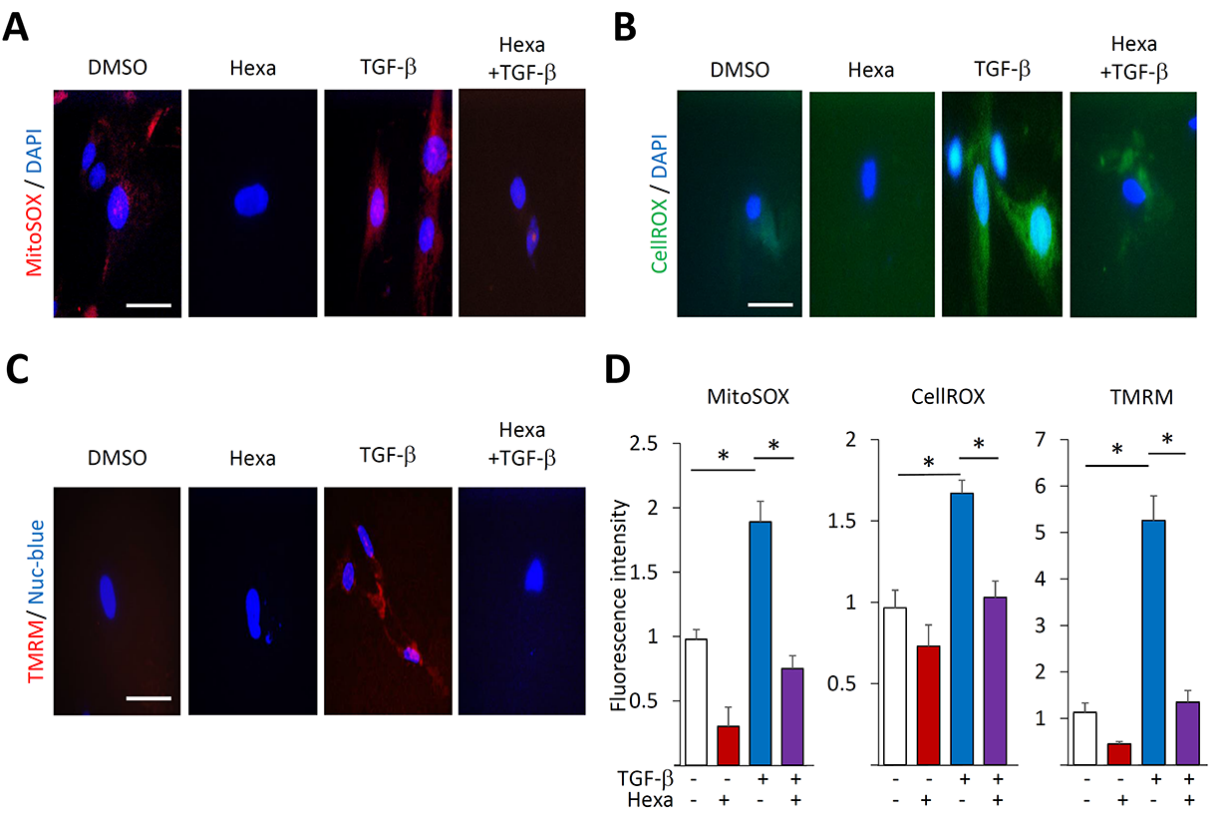
Hexafluoro abrogates TGF-β-induced ROS production and mitochondrial membrane potential Lung fibroblasts were preincubated with 10 μM hexafluoro for 30 min, followed by TGF-β2 (10 ng/ml) for 24 h. **A.**, **B.** Mitochondrial ROS (red) determined using MitoSOX Red Mitochondrial Superoxide indicator, and cytosolic ROS (green) determined using CellROX Green. Cells were counterstained with DAPI (blue). Bar = 10 μm. **C.** Cultures stained with Nuc-blue (blue) and TMRM fluorescent dyes (red). Bar = 10μm. **D.** Quantification of MitoSOX, CellROX and TMRM fluorescence. Bars represent means ±SD of triplicate determinations. *p < 0.05.

Myofibroblast differentiation in response to TGF-β is associated with, and indeed dependent on, cytosolic as well as mitochondrial ROS production and changes in mitochondrial membrane potential [29, 30]. We therefore examined the effects of hexafluoro on alterations in mitochondrial function in the context of TGF-β-induced myofibroblast differentiation. As expected, TGF-β treatment resulted in enhanced mitochondrial ROS production, which was markedly attenuated when fibroblasts were pre-treated with hexafluoro (Figure 5A). Enhanced cytosolic accumulation of ROS was similarly attenuated (Figure 5B). Next, fibroblasts were loaded with TMRM, a mitochondrial membrane potential-sensitive fluorescent dye. While treatment with TGF-β was associated with the appearance of perinuclear red punctae that are indicative of active polarized mitochondria, the uptake of TMRM was substantially attenuated in the presence of hexafluoro (Figure 5C, 5D). Taken together, these results indicate that in TGF-ß-treated normal fibroblasts hexafluoro exerts mitochondrial-protective effects.

### Hexafluoro blocks activation of Smad2/3 and Stat3

**Figure 6:**
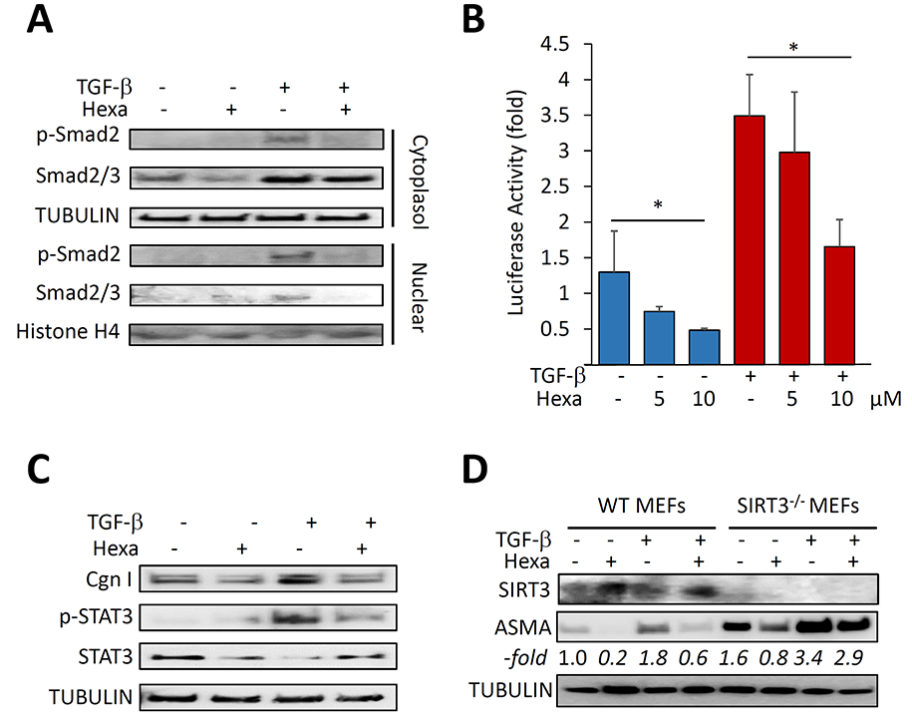
Hexafluoro abrogates fibrotic signaling **A.**, **C.** Lung fibroblasts were treated with 10 μM hexafluoro in the presence or absence of TGF-β (10ng/ml) for 120 min. **A.** Cytoplasmic lysates and nuclear extracts were examined by Western analysis. Representative immunoblots. **B.** NHLF transiently transfected [SBE]_4_-luc along with SIRT3 or empty vector, were pre-incubated with 10 μM hexafluoro for 30 min, followed with TGF-β (10 ng/ml) for 24 h. Whole cell lysates were analyzed for their luciferase activities. Results are the means ±SD of triplicate experiments. *p < 0.05. **C.** Whole cell lysates were examined by Western analysis. Representative immunoblots. Cgn I, Type I collagen. **D.** Confluent SIRT3-null (SIRT3^−/−^) and wild-type (WT) embryonic fibroblasts (MEFs) in parallel were preincubated with hexafluoro (10 μM) for 30 min, followed by TGF-β (10 ng/ml) for 24 h. Whole cell lysates were examined by Western analysis. Band intensities normalized to tubulin are shown as -fold change below.

To investigate the mechanism underlying the anti-fibrotic effects of hexafluoro, we focused on the canonical Smad signal transduction pathway fundamental for fibrotic responses [31]. We found that Smad2 phosphorylation and nuclear translocation induced by TGF-β in normal fibroblasts were markedly attenuated by hexafluoro (Figure 6A). Moreover, the magnitude of Smad-dependent transcriptional responses was reduced in a dose-dependent manner (Figure 6B). Hexafluoro also attenuated TGF-β-induced phosphorylation of STAT3, a key mediator of fibrotic IL-6 signaling [32] and a potential effector in pathological fibrosis (Figure 6C). To examine the cell-autonomous role of SIRT3 as a negative regulator mediating the anti-fibrotic effects of hexafluoro, we examined fibrotic responses in fibroblasts lacking endogenous SIRT3. The results indicated that both basal and TGF-β-stimulated levels of αSMA were elevated in SIRT3^−/−^ MEFs. Moreover, these cells were partially resistant to the anti-fibrotic effects of hexafluoro, indicating that these inhibitory effects were at least in part SIRT3-dependent (Figure 6D).

### Hexafluoro ameliorates experimentally-induced organ fibrosis in mouse

**Figure 7:**
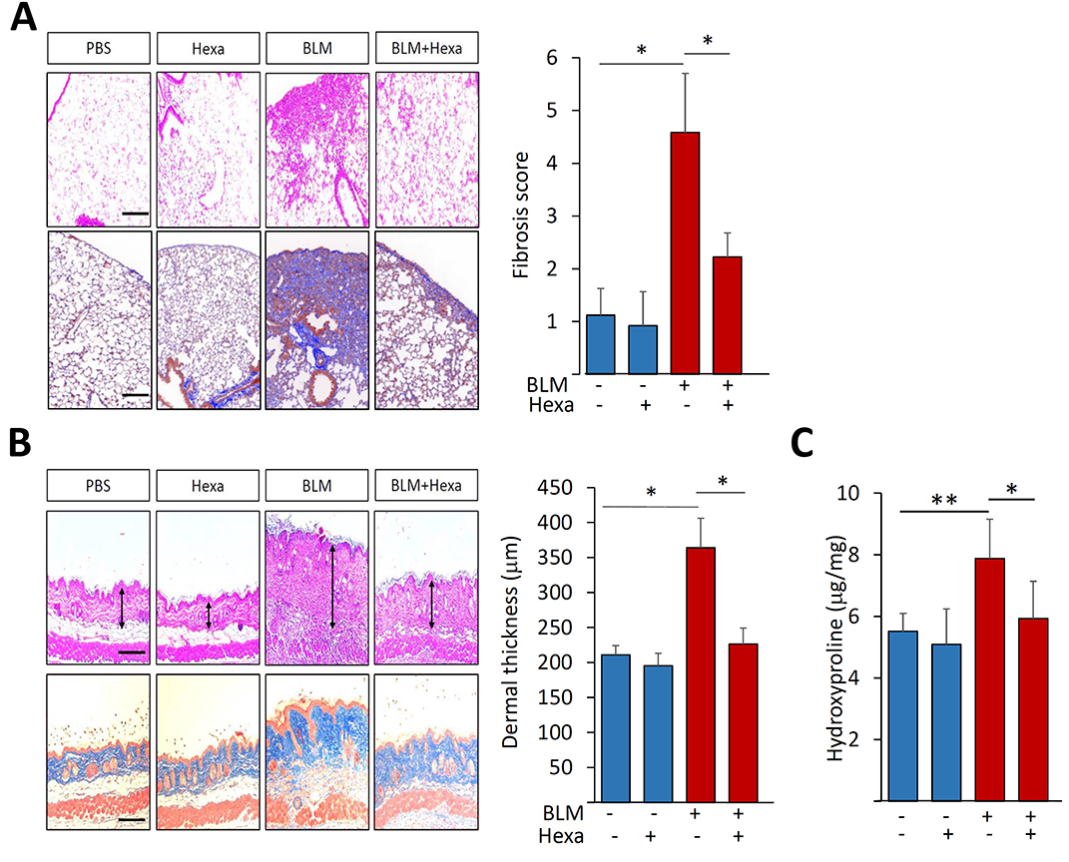
Hexafluoro treatment ameliorates bleomycin-induced organ fibrosis Mice were treated with s.c. bleomycin (BLM) alone for 14 days, or in combination with i.p. hexafluoro, and sacrificed on day 28. **A.** Lung changes. Left panels, H&E (upper) and Trichrome (lower) stain. Original magnification X 100. Right panels, lung fibrosis score determined as described in Materials and Methods. Bars are the means ± SD of each group (4-7 mice/group). *p < 0.05. **B.** Attenuation of dermal fibrosis. Left panels, H&E (upper) and Trichrome (lower) stain. Bar = 50 μm. Right panel, quantification of dermal thickness. Bars represent means ± SEM (4-7 mice per group). *p < 0.05. **C.** Collagen content of the skin determined by hydroxyproline assays. Results are means ± SD of duplicate determinations from 4-7 mice per group; *p < 0.05; **p < 0.01

In light of the robust, and at least partially SIRT3-dependent, anti-fibrotic effects of hexafluoro in fibroblasts, it was of interest to assess the impact of hexafluoro treatment in a bleomycin-induced model of multiple-organ fibrosis [33]. Female 8-10 week-old C57BL/6J mice were randomized to treatment with vehicle, s.c. bleomycin, or a combination of s.c. bleomycin plus i.p. hexafluoro (70 mg/kg/day). In these experiments, hexafluoro for up to 28 days was well tolerated, and treatment was not associated with significant weight loss, behavioral changes or other signs of toxicity. Bleomycin given s.c. induced prominent changes in the lungs (Figure 7A). Pulmonary fibrotic pathology was substantially attenuated in mice treated with hexafluoro (Figure 7A, right panel). Bleomycin-treated mice also developed skin fibrosis and increased collagen accumulation that were significantly ameliorated by hexafluoro (Figure 7B, 7C). Together, these results showed that hexafluoro has potent protective effects on both lung and skin fibrosis in a bleomycin-induced mouse model.

### SIRT3 expression and function are decreased in SSc and in fibrotic tissues in the mouse

**Figure 8:**
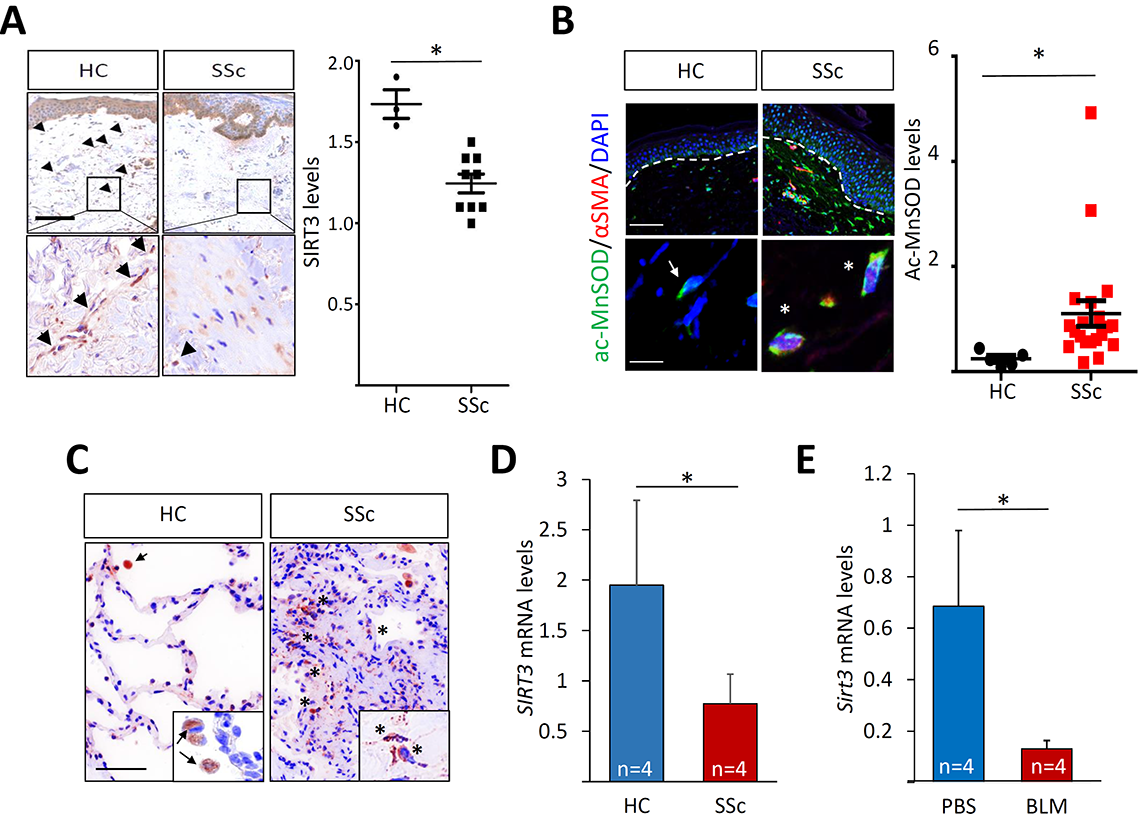
Attenuated SIRT3 expression and activity in SSc and in mouse models of fibrosis **A.** Immmunohistochemistry of skin biopsies. Healthy control (*n* = 3) and dcSSc (*n* = 9) skin biopsies were evaluated using antibodies to SIRT3. Left panels, representative images. Arrowheads indicate immunopositive spindle-shaped cells within the dermis. Lower panels, higher magnification of boxed areas. Bar = 12.5 μm. Right panel, SIRT3 scores calculated as described in Materials and Methods. Bars represent the means ±SD; *p* < 0.05. **B.** Immunfluorescence. Left panels, immunostain of healthy control (*n* = 5) and SSc (*n* = 20) skin biopsies. Representative images. Green, Ac-MnSOD; red, αSMA; blue, DAPI. White dashed lines indicated epidermis-dermis junction. Arrows, Ac-MnSOD positive cells; star, Ac-MnSOD and αSMA double-positive cells. Bar = 100 μm (upper panels) and 10 μm (lower panels). Right panel, Ac-MnSOD levels. Each dot represents a biopsy. **C.** Immunohistochemistry of control (*n* = 3) and SSc (*n* = 6) lung biopsies using anti-Ac-MnSOD. Representative images. Inset, higher magnification. Arrow, Ac-MnSOD-positive alveolar macrophages; asterisk, Ac-MnSOD-positive stromal cells. Bar = 50 μm. **D.**, **E.** Real-time qRT-PCR of lesional skin. Results, normalized with *GAPDH*, and the means ± SD of each group. *p < 0.05. D. RNA from confluent control (*n* = 4) and SSc (*n* = 4) skin fibroblasts was subjected to real-time qPCR. E. Mice treated with s.c. bleomycin (BLM; *n* = 4) or PBS (*n* = 4) for 14 days in parallel were sacrificed on day 28. RNA isolated from lesional skin was analyzed by qRT-PCR. Results, normalized with *Gapdh*, are means ± SD of each group. *p < 0.05.

The potent *in vitro* and *in vivo* anti-fibrotic effects of hexafluoro led us to speculate that its target, SIRT3, itself might play a regulatory role in pathological fibrosis in SSc, and its dysfunction might contribute to disease progression. To address this possibility, we investigated SIRT3 expression and activation in SSc biopsies. Demographic and clinical features of the subjects are shown in Table 1 and Table 2. By immunohistochemistry, SIRT3 expression was detectable in normal skin biopsies, and appeared to be primarily localized in spindle-shaped fibroblastic cells, as well as in round cells throughout both the papillary and reticular dermis (Figure 8A). Additionally, prominent SIRT3 expression was evident in keratinocytes in the basal epidermis. While SSc skin biopsies generally demonstrated retention of SIRT3 in the basal epidermis, within the dermis substantially lower SIRT3 levels were observed in SSc compared to healthy control skin biopsies (*P* < 0.05). Of note, we found a significant negative correlation (R^2^ = 0.5421; *p* = 0.04) between the SIRT3 score (a semi-quantitative measure of SIRT3 levels) within the lesional skin and disease duration. In contrast, the modified Rodnan Skin Score (MRSS), a global measure of skin induration, showed no significant correlation with the skin SIRT3 score. To evaluate SIRT3 activity, levels of acetylated MnSOD, the prototypic SIRT3 substrate and a sensitive marker of SIRT activity, were examined. In sharp contrast to healthy skin biopsies, in SSc skin biopsies in a majority of interstitial cells within the dermis were immunopositve for Ac-MnSOD (Figure 8). Many of these cells were also positive for the myofibroblast marker αSMA, indicating attenuated SIRT3 activity within lesional myofibroblasts. Lung biopsies from patients with SSc-ILD (*n* = 5, Table 3) showed elevated expression of Ac-MnSOD within the fibrotic parenchyma compared to control lungs (*n* = 3). Alveolar macrophages showed detectable Ac-MnSOD expression in both SSc and control lungs.

**Table 1:** Clinical features of subjects donating skin biopsies used for SIRT3 immunohistochemistry

	Age yrs	gender	MRSS	Skin score at biopsy site	Disease duration from 1^st^ non-RP manifestation (month)
SSc 1	49	F	3	0	44
SSc 2	51	M	48	3	9
SSc 3	50	F	24	2	101
SSc 4	34	F	32	2	40
SSc 5	57	F	11	1	64
SSc 6	50	F	9	1	8
SSc 7	70	F	4	0	9
SSc 8	57	F	9	0	47
SSc 9	43	F	2	0	1

MRSS, modified Rodnan skin score.

RP, Raynaud's phenomenon.

Skin score at biopsy site scored as follows: 0, normal thickness; 1, mild thickening; 2, moderate thickening; 3, severe thickening.

**Table 2:** Clinical features of subjects donating skin samples used for Ac-MnSOD immunofluorescence

	Age	gender	MRSS	Skin score at biopsy site	Disease duration from 1^st^ non-RP manifestation (month)
SSc 10	67	F	10	0	23
SSc 11	48	M	10	0	20
SSc 12	39	M	13	1	13
SSc 13	50	F	14	1	23
SSc 14	52	F	17	1	18
SSc 15	55	M	19	2	33
SSc 16	52	F	24	2	37
SSc 17	58	F	3	0	124
SSc 18	62	F	3	0	140
SSc 19	30	F	1	0	71
SSc 20	56	F	6	0	30
SSc 21	19	F	0	0	16
SSc 22	56	F	3	0	104
SSc 23	36	F	2	0	151
SSc 24	57	F	4	0	124
SSc 25	48	F	5	0	240
SSc 26	56	M	2	0	28
SSc 27	31	F	25	3	49
SSc 28	51	F	35	2	11
SSc 29	69	M	41	3	10

MRSS, modified Rodnan skin score.

RP, Raynaud phenomenon.

Skin score at biopsy site scored as follows: 0, normal thickness; 1, mild thickening; 2, moderate thickening; 3, severe thickening.

**Table 3:** Clinical features of subjects providing lung samples used for Ac-MnSOD immunofluorescence studies

	S-15-7641	S15-47085	S15-39544	S16-11379
Age (yrs)	61	42	58	53
Sex	F	M	F	M
Specimen	Explant	Explant	Explant	Explant
Duration of disease	Long standing	Long standing	Long standing	Long standing
Predominant histological pattern	NSIP	NSIP and UIP	NSIP and UIP	UIP

NSIP, nonspecific interstitial pneumonitis; UIP, usual interstitial pneumonitis.

When propagated in culture, explanted SSc fibroblasts maintain their activated phenotype even in the absence of exogenous stimuli, due in part to cell-autonomous TGF-β receptor phosphorylation and activity [34, 35]. To examine the cell-autonomous expression of SIRT3 in SSc, fibroblasts from dcSSc (*n* = 4) and matched healthy control (*n* = 4) skin biopsies were studied in parallel. By qPCR, significantly reduced *SIRT3* expression was noted in SSc fibroblasts (*p* = 0.04; Figure 8D). Incubation of SSc fibroblasts (*n* = 4) with hexafluoro was able to attenuate *COL1A1*, *αSMA*, and *FN^EDA^* expression (Table 4). We next examined *Sirt3* expression in a murine model of scleroderma induced by s.c. bleomycin. Development of skin fibrosis in these mice was accompanied by significant down-regulation of *Sirt3* within the lesional dermis (Figure 8E). Taken together, these results indicate that SIRT3 levels and activity are impaired in fibrotic lesions and explanted skin fibroblasts in SSc, as well as in a mouse model of scleroderma.

**Table 4:** Hexafluoro mitigates fibrotic gene expression in both normal SSc skin fibroblasts

Cell line	COL1A1*	ASMA*	FN^EDA^*
S1	0.30	0.36	0.47
S2	0.56	0.07	0.95
S3	0.35	0.37	0.27
S4	0.30	0.36	0.67

* fold change in mRNA levels compared to untreated cultures.

Confluent cultures of low-passage SSc skin fibroblasts (*n* = 4) were incubated with hexafluoro (10 μM) for 24 h. Results of real-time qPCR, normalized with GAPDH.

## DISCUSSION

We show here that SIRT3, the major mitochondrial deacetylase, has potent cell-intrinsic anti-fibrotic effects, and its expression and activity are reduced in fibrotic tissues and explanted skin fibroblasts from patients with SSc. Hexafluoro, a novel synthetic honokiol derivative, induces SIRT3 expression and activity, and mitigates TGF-ß-induced mitochondrial oxidative stress and fibroblast stimulation, at least in part via stimulation of cellular SIRT3. Moreover, hexafluoro treatment has striking effects on mitigating skin and lung fibrosis in bleomycin-treated mice.

There is growing recognition of the importance of mitochondrial ROS as mediators of TGF-β signal transduction in the context of fibrogenesis [29, 36]. SIRT3 plays critical role in regulating mtROS production [25, 37]. Here we examined the regulation of SIRT3 expression and function by TGF-β, the major fibrogenic growth factor implicated in fibrosis [4, 31]. Our results showed that expression of SIRT3 is suppressed by TGF-ß treatment of explanted skin and lung fibroblasts. A series of cell-based assays with gain- and loss-of-function were carried out to delineate the mechanism of SIRT3 effects in the context of fibrogenesis. Ectopic SIRT3 attenuated TGF-β induced stimulation of collagen synthesis and fibrotic responses in both lung and skin fibroblasts, while RNAi of cellular SIRT3 resulted in enhanced fibrotic responses. The anti-fibrotic effects of SIRT3 involved disruption of canonical TGF-ß signal transduction and Smad-dependent transcription. Furthermore, we found that both tissue expression and function of SIRT3 were substantially reduced in skin and lung biopsies from SSc patients, as well as in mice with bleomycin-induced skin fibrosis. Taken together, these findings suggest that SIRT3 might have an important negative regulatory function to check TGF-β-dependent fibrotic responses in fibroblasts, and its deficiency in SSc, possibly reflecting its down-regulation by TGF-β within the fibrotic milieu, might play a causal role in the persistence of fibrosis in these tissues. We did not demonstrate correlation between SIRT3 expression levels in the skin and the extent of skin fibrosis, as measured by MRSS. Whether reduced SIRT3 expression/activity might correlate with progression of skin fibrosis over time, suggesting a potential role as a predictive biomarker, remains to be determined in longitudinal analyses.

Sirtuins belong to an evolutionary conserved family of NAD^+^-dependent protein lysine deacylases that play key roles in the regulation of metabolism, stress responses, and aging processes. Honokiol, a naturally-occuring biphenolic compound, has pleiotropic effects and potential pharmacological utility [38]. To further enhance honokiol's drug-like properties, recent efforts focused on modification resulting in the novel compound hexafluoro [24]. We now show that hexafluoro induced a rapid increase in SIRT3 mRNA and protein expression, and rescued SIRT3 expression even in the presence of TGF-β. Stimulation of SIRT3 was accompanied by attenuation of cytosolic and mitochondrial ROS, and restoration of perturbed mitochondrial membrane potential. These mitochondrial-protective effects of hexafluoro are likely to be related to induction of cellular SIRT3, which serves as the major mitochondrial deacetylase to reduce oxidative stress [12, 39]. However, the effects of honokiol derivatives appear to be cell-type specific, and ROS production was enhanced by honokiol derivatives, including hexafluoro, in melanoma cell lines [24]. In our studies, treatment of both lung and skin fibroblasts with hexafluoro attenuated TGF-β induced pro-fibrotic gene expression and myofibroblast differentiation, contractility and migration, and mitigated constitutive fibrotic gene expression in SSc fibroblast. These effects were associated with attenuated Smad signaling, indicating that hexafluoro induced factors that antagonize receptor-dependent Smad activity. Activation of Stat3, an important mediator of cytokine signaling linked to fibrosis, was also attenuated [40]. Whether impaired Stat3 activation in fibroblasts treated with hexafluoro is a direct effect, or is mediated via endogenous SIRT3, remains to be addressed. Treatment of mice with hexafluoro mitigated both lung and skin fibrosis induced by bleomycin. The role of attenuated mitochondrial oxidative stress and altered autoimmunity, in these anti-fibrotic effects and if they remain to be established.

In summary, we show here that the mitochondrial deacetylase SIRT3 has cell-intrinsic potent anti-fibrotic properties in fibroblasts, and its expression and activity are impaired in SSc skin and lung biopsies. A recent study demonstrated comparable reduced SIRT3 expression in the explanted lungs from patients with SSc-ILD, providing further evidence for the potential role of impaired SIRT3 function in the disease [41]. Furthermore, the novel synthetic honokiol derivative hexafluoro has SIRT3 agonist activities coupled with inhibitory effects on mitochondrial ROS generation and TGF-ß-induced fibrotic responses in fibroblasts, and on experimental organ fibrosis in mice. The results implicate, for the first time, SIRT3 deficit as a pathogenic mechanism underlying fibrosis in SSc, and suggest that hexafluoro might have therapeutic potential via restoring SIRT3 in the treatment for fibrotic conditions.

## MATERIALS AND METHODS

### Human subjects

The protocols for human tissue procurement (skin and lung) were approved by the Institutional Review Boards for Human Studies at Northwestern University. All patients fulfilled the American College of Rheumatology/European league Against Rheumatism 2013 classification criteria for SSc [42]. Skin biopsies were performed after obtaining written informed consent. Lung tissues were obtained from five SSc patients undergoing lung transplantation for advanced ILD. The demographics and clinical characteristics of patients were listed in Tables 1, 2 and 3.

### Bleomycin-induced scleroderma in the mouse

Animal protocols were institutionally approved by the Animal Care and Use Committees of Northwestern University. Eight to ten week-old female C57/BL6J mice (The Jackson Laboratory, Bar Harbor, ME) were randomized to receive vehicle, bleomycin (APP, Schaumburg, IL), or a combination of hexafluoro and bleomycin (4-7 mice/group). Hexafluoro was synthesized and purified by column chromatography as described [24]. Mice were given daily subcutaneous (s.c.) injections of PBS or 10 mg/kg bleomycin for 14 days [43]. Hexafluoro was given by daily intraperitoneal (i.p.) injections five times/week at a dose of 70 mg/kg in a cocktail made by dissolving 16 mg of compound into 100 μl of absolute ethanol. The ethanol-compound solutions were then added to 20% soy-fat Intralipid (Frensenius Kabi, Lake Zurich, IL) and vortexed vigorously [24]. Mice were sacrificed on day 28, and lesional skin and lung were harvested. Each experimental group included 4-7 mice. Full-thickness skin sections were taken from injected back region, and lung sections were taken from the right lungs. Tissues were fixed in 10% formaldehyde for 48 or 96 h respectively, and embedded in paraffin. Sections were stained with hematoxylin and eosin (H&E) or Masson's trichrome. Dermal thickness, defined as the distance from the epidermal-dermal junction to the junction between the dermis and subcutaneous fat, was determined at five random locations per section [28]. All sections were examined independently by two investigators in a blinded manner. Severity of lung fibrosis was graded on a scale of 0 to 8 by examining five randomly chosen fields of the same right lobe at 100 x magnification as follows: grade 0, normal lung; grade 1, minimal fibrous thickening of alveolar or bronchiolar walls; grade 3, moderate thickening of walls without obvious damage to lung architecture; grade 5, increased fibrosis with definite damage to lung structure and formation of fibrous bands or small fibrous masses; grade 7, severe distortion of structure and large fibrous areas; and grade 8, total fibrous obliteration. Sections were scored independently by two investigators in a blinded manner. Collagen accumulation was determined by measuring hydroxyproline content in half of 8-mm skin biopsy samples [44]. Results are expressed as total hydroxyproline per mg tissue.

### Fibroblast cultures

Primary cultures of dermal fibroblasts were established by explanation from foreskins from healthy newborns or from skin biopsies from SSc patients or age-matched healthy controls [43]. Normal adult lung fibroblast were purchased from LONZA (Walkersville, MD). Cells were incubated at 37°C in an atmosphere of 5%CO_2_ in Dulbecco's modified Eagle's medium (DMEM) supplemented with 10% fetal bovine serum, 1% vitamins, 1% penicillin/streptomycin, and 2 mM L-glutamine (all from BioWhittaker) for skin fibroblasts and FGM-2 bullet kit (LONZA) for NHLF, respectively. Cells were used between passage 4 and 8. At early confluence, cultures were incubated with hexafluoro in the presence or absence of TGF-β2 (10 ng/ml) (PeproTech, Rocky Hill, NJ). Cell toxicity was evaluated using lactate dehydrogenase cytotoxicity assay kits (BioVision, Milpitas, CA), and cell viability by trypan blue dye exclusion.

### Gel contraction and cell migration assays

To assess the effects of hexafluoro on cell contraction, lung fibroblasts were seeded in type I collagen gels (BD Bioscience, San Jose, CA) that were then incubated in medium with or without TGF-β2 (10 ng/ml) and hexafluoro for up to 72 h [43]. After 72h, gel diameters were determined. Modulation of cell migration by hexafluoro was evaluated by *in vitro* wound healing assays. Briefly, confluent monolayers of fibroblasts were incubated in serum-free medium with hexafluoro for 12 h in the presence of 10 μg/ml Mitomycin C (Sigma, St Louis, MO), and scratch wounds were created using standard p1000 pipette tips [43]. Cell migration was then monitored by phase-contrast microscopy for up to 48 h. Gap width was determined at 3 different sites per sample at indicated intervals.

### Plasmids, small interfering RNA (siRNA), and transient transfection assays

Small interfering RNAs specific for SIRT3 and scrambled control siRNA were purchased from Santa Cruz Biotechnology (Dallas, TX). The plasmid SBE_4_-TK-Luc contains four copies of the consensus Smad-binding element linked to thymidine kinase and luciferase genes [43]. Subconfluent cultures were transfected with SIRT3 expression vector (OriGene, Rockville, MD) or empty vector, SBE_4_-TK-Luc or siRNA (Santa Cruz) for 48 h, followed by incubation with or without TGF-β2 (10 ng/ml) and hexafluoro for 24 h. Cultures were harvested and whole-cell lysates were assayed for their luciferase activities using the Dual-Luciferase Reporter Assay system (Promega, Madison, WI) [43]. The reporter vector pRL-TK *Renilla* luciferase (pRL-TK Luc) was used in each experiment as an internal control, and experiments were repeated at least 3 times.

### Evaluation of ROS generation and mitochondrial membrane potential changes

At early confluence fibroblasts seeded on cover slips were incubated in media with or without TGF-β2 (10 ng/ml) or hexafluoro (10 μM) for 24 h. Mitochondrial ROS levels were determined using MitoSOX Red superoxide indicator reagent that concentrates within mitochondria and fluoresces red when oxidized by ROS. Cytosolic ROS production was assessed using CellROX green reagent (both from Invirogen). Fluorescence was determined under a confocal microscope (Nikon C2+, Nikon Tokyo, Japan) at 495 nm (green), 565 nm (red) and 400 nm (blue), and intensity was quantitated using Image J. In order to assess changes in mitochondrial membrane potential, tetramethylrhodamine (TMRM) mitochondrial membrane potential-dependent cationic red dye (Thermo Scientific) was used [30].

### RNA isolation and quantitative real-time PCR

Total RNA was isolated from explanted fibroblasts using Quick RNA Miniprep (Zymo Research), and quantitative PCR was performed [43]. Levels of mRNA normalized to *GAPDH* levels in each sample were determined by calculating 2^−ΔΔ^C_t_^.^ Dissociation analysis for each primer pair and reaction was performed to verify specific amplification.

### Western analysis

At the end of the experiments, cells were lysed with passive protein lysis buffer (Promega, Fitchburg, WI), and 5-15μg total proteins were applied to 4-15% SDS-polyacrylamide gel. After electrophoresis, the proteins were transferred to PVDF membranes, followed by blocking in the buffer containing 10% fat-free dry milk. The membranes were probed with indicated first antibodies overnight, and then washed three times in TBS-T and incubated with HRP-conjugated secondary antibodies for 60 min and visualized using chemiluminescence ECL reagent (Sigma). The following antibodies were used: anti-tubulin, anti-SIRT3, anti-SIRT1, anti-histone H4, anti-Fn^EDA^, anti- αSMA (Sigma), anti- type I collagen (Southern Bio), anti-phospho-Smad2, anti-Smad2/3, anti-STAT3, and anti-phospho-STAT3 (Cell Signaling). Membranes were developed using Fuji scanner and analyzed by AIDA software (Fuji, Inc., Tokyo, Japan).

### Immunohistochemistry and immunofluorescence

Skin and lung biopsies from healthy adults, non-SSc subjects and SSc patients (Tables 1 and 2) were examined by immunohistochemistry. Briefly, 4-μm paraffin-embedded sections were incubated overnight with primary antibodies to SIRT3 (Cell Signaling Technology, San Antonio, TX) or Ac-MnSOD (Abcam, Cambridge, MA) overnight, followed by incubation with biotinylated donkey anti-rabbit secondary antibodies and streptavidin-linked alkaline phosphatase (AP) (Jackson ImmunoResearch, West Grove, PA) or AlexaFlouro conjugated secondary antibodies (Invitrogen). Levels of SIRT3 were evaluated using a semi-quantitative approach. Immunostaining intensities were determined by two independent blinded observers by scoring ≥ 60 individual fibroblastic cells in 3 randomly chosen high-power fields per biopsy throughout the dermis as follows: 0 = no detectable staining: 1 = faint staining: 2 = moderate staining: 3 = strong staining. Levels of Ac-MnSOD in dermal cells were quantitated by measuring fluorescence intensity using ImageJ, and the results were normalized with DAPI fluorescence intensity. SIRT3 and ac-MnSOD scores were calculated by taking the means of fluorescence intensities. Isotype IgG was used as negative controls (Suppl. Fig 3). To evaluate cellular levels of type I collagen and αSMA, confluent fibroblasts were pre-incubated with 10 μM hexafluoro for 60 min prior to incubation with 10 ng/mL TGF-β2. Twenty-four hours later, cells were fixed, incubated with primary antibodies against type I collagen (1:200; Southern Biotech, Birmingham, AL) or α-SMA (1:500; Sigma Aldrich) overnight, followed by incubation with Alexa Fluor 594-conjugated donkey anti-goat (Invitrogen) or Alexa Fluor 488-conjugated chicken anti-mouse antibodies for 120 min. Nuclei were identified by DAPI staining (1:5000). Nonimmune IgG was used as a negative control in each experiment. Following stringent washing, slides were examined under Nikon C2+ confocal microscope. Each experiment was repeated at least 3 times with consistent results.

### Statistical analysis

All values are expressed as the mean ±SD. At least three replicates were applied for each experiment. Statistical differences among groups were determined using either Student's *t* test or one-way ANOVA. In the animal and human studies, differences between the groups were evaluated using non-parametric Mann–Whitney U test. *P* values of less than 0.05 were considered statistically significant.

## SUPPLEMENTARY MATERIAL


