# Supplementary material for: SIRT3 is attenuated in systemic sclerosis skin and lungs, and its pharmacologic activation mitigates organ fibrosis
Source: Oncotarget. 2016 Oct 6;7(43):69321–36. doi: 10.18632/oncotarget.12504 (PMC5342480; doi:10.18632/oncotarget.12504)
Supplement: Supplementary file 1 [file oncotarget-07-69321-s001.pdf]

SIRT3 is attenuated in systemic sclerosis skin and lungs, and its pharmacologic activation mitigates organ fibrosis

Supplementary Material

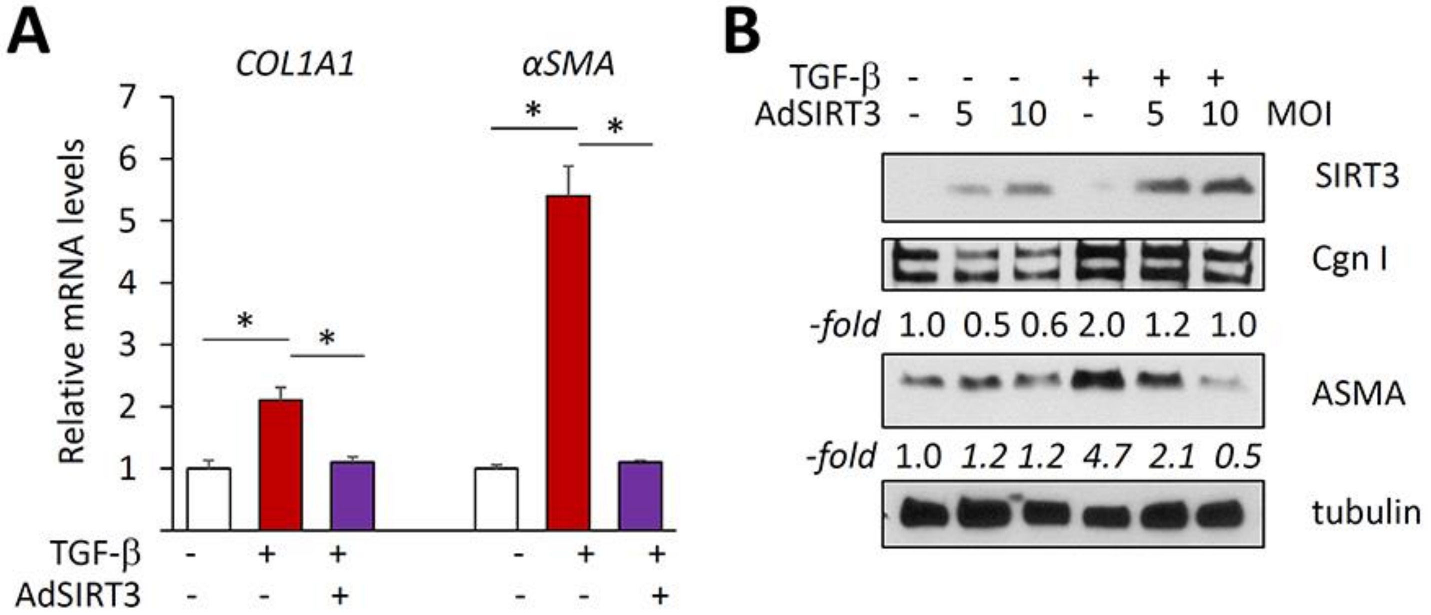

Supplementary Figure 1. SIRT3 negatively regulates fibrotic responses in skin fibroblasts. Normal human skin fibroblasts were infected with SIRT3 adenovirus (10 MOI or as indicated), and incubated with TGF-β2 (10 ng/ml) for 24 h. **A.** qRT-PCR. Results normalized with *GAPDH* are means ± SD of triplicate determinations of an experiment representative of three. *p<0.05. **B.** Western immunoblot of whole cell lysates. Images representative of duplicate experiments. -fold change in band intensities normalized for tubulin in each lane shown below. MOI, multiplicity of infection. Cgn I, Type I collagen.

\

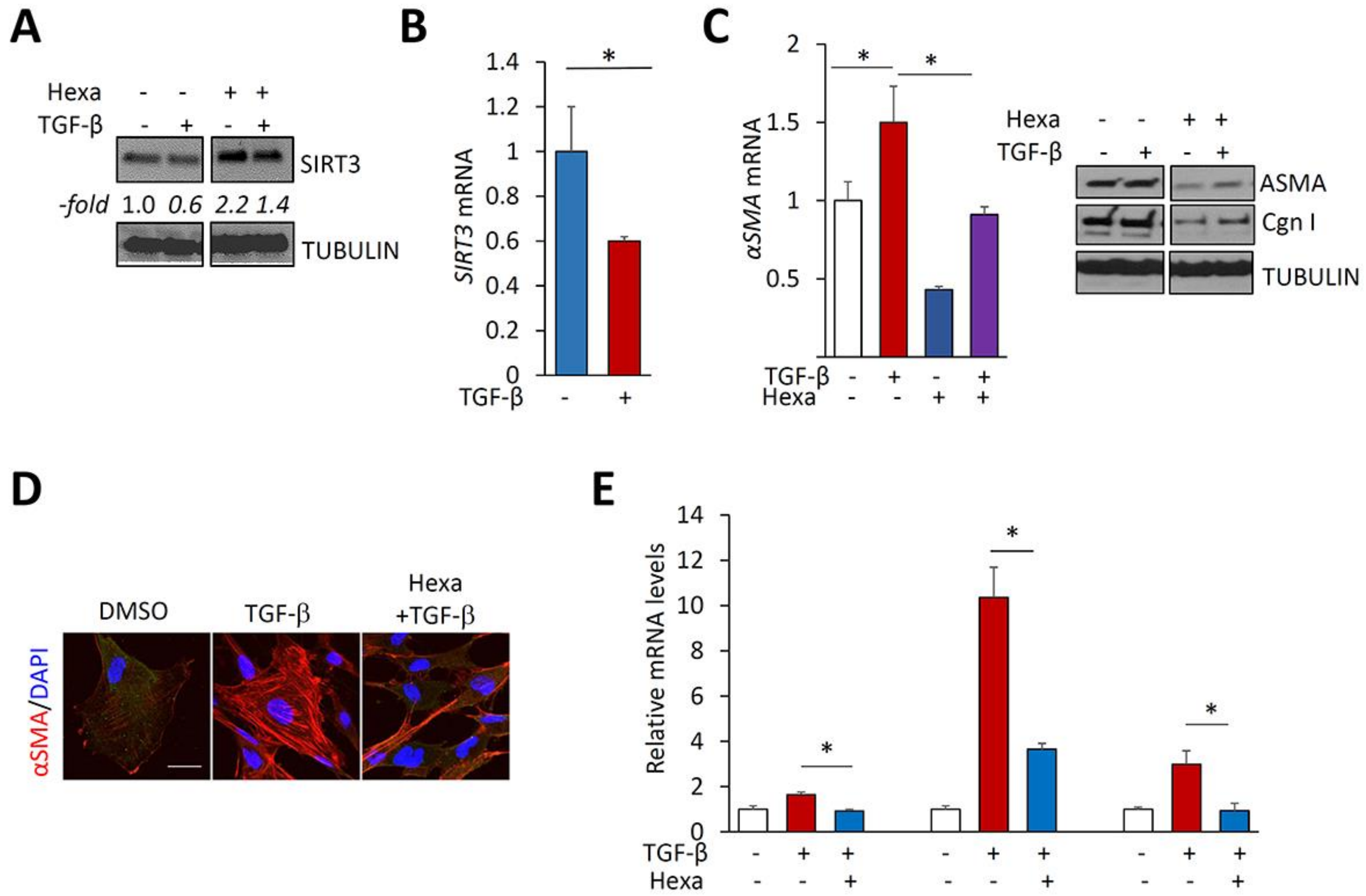

Supplementary Figure 2. Hexafluoro abrogates basal and stimulated fibrotic gene expression in skin fibroblasts. Human skin fibroblasts were preincubated with hexafluoro (10 µM) for 30 min prior to TGF-ß (10 ng/ml) for 24 h, or hexafluoro was added to the cultures 24 h after TGF-β (**E**). **A.** Western analysis. Representative images. -fold change in protein levels shown below. **B, E.** Real-time qPCR. Results are means + SD of triplicate determinations from an experiment representative of three; *p<0.05. **C.** Left panel, real-time qPCR results normalized with *GAPDH*. Means ± SD of triplicate determinations. *p<0.05. Right panels, Western analysis of whole cell lysates. Representative images. Cgn I, Type I collagen. **D.** Immunofluorescence confocal microscopy with antibodies to αSMA. Nuclei identified with DAPI. Representative images; bar =10 µm.

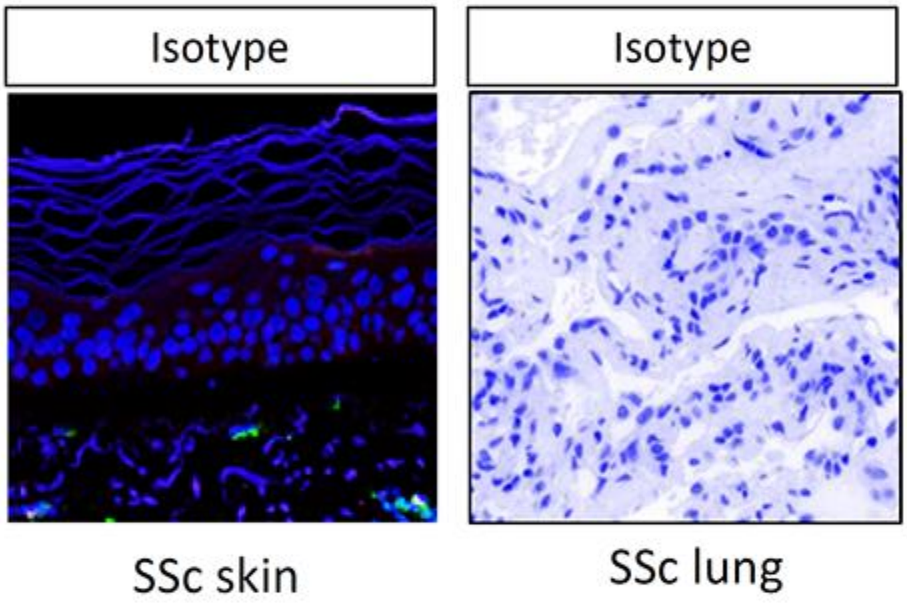

Supplementary Figure 3. Isotype controls in skin and lung biopsies. Isotype control IgG was used to replace primary Ac-MnSOD antibody; left, representative IF images in skin biopsies; right, IHC images of lung biopsies.
